# Macular thickness measured via optic coherence tomography in healthy adults aged 45 years and older: The Brazilian Amazon Region Eye Survey

**DOI:** 10.5935/0004-2749.2024-0411

**Published:** 2025-09-10

**Authors:** Sung Eun Song Watanabe, Adriana Berezovsky, Arthur Gustavo Fernandes, Bruna Ferraço Marianelli, João Marcello Furtado, Marcela Cypel, Paulo Henrique Morales, Marcos Jacob Cohen, Cristina Coimbra Cunha, Márcia Higashi Mitsuhiro, Galton Carvalho Vasconcelos, Mauro Campos, Nívea Nunes Ferraz, Paula Y. Sacai, Jacob Moysés Cohen, Sergio Muñoz, Rubens Belfort Jr., Solange Rios Salomão

**Affiliations:** 1 Departamento de Oftalmologia e Ciências Visuais, Escola Paulista de Medicina, Universidade Federal de São Paulo, São Paulo, SP, Brazil; 2 Departamento de Oftalmologia, Otorrinolaringologia e Cirurgia de Cabeça e Pescoço, Faculdade de Medicina de Ribeirão Preto, Universidade de São Paulo, Ribeirão Preto, SP, Brazil; 3 Divisão de Oftalmologia, Departamento de Cirurgia, Faculdade de Medicina, Universidade Federal do Amazonas, Manaus, AM, Brazil; 4 Departamento de Oftalmologia e Otorrinolaringologia, Faculdade de Medicina, Universidade Federal de Minas Gerais, Belo Horizonte, MG, Brazil; 5 Departamento de Salud Publica, Universidad de La Frontera, Temuco, Chile

**Keywords:** Retinal diseases/diagnosis, Macula lutea/pathology, Macular degeneration/diagnosis, Diabetic retinopathy/diagnosis, Vision, low, Vision tests, Tomography, optical coherence/methods, Young adult, Cross-sectional studies, Brazil/epidemiology

## Abstract

**Purpose:**

This study evaluated macular thickness using spectral-domain optical coherence tomography in healthy participants from a population-based eye survey.

**Methods:**

The Brazilian Amazon Region Eye Survey was a population-based study assessing the prevalence and causes of visual impairment, blindness, and ocular diseases in adults aged ≥45 years from urban and rural areas of Parintins. A subgroup was selected based on inclusion criteria for both eyes: best-corrected visual acuity ≥20/32, normal eye examination results, and no prior ocular surgery. Scans were performed using the iVue optical coherence tomography device. Measurements were taken from the nine subfields defined by the Early Treatment Diabetic Retinopathy Study, examining the full retina as well as the inner and outer retinal layers. Associations of retinal thickness with age and sex were also analyzed. Statistical significance was set at p≤0.05.

**Results:**

In total, 70 healthy participants (25 males), aged 45–65 years (mean=52 ± 5), were included. Mean central foveal thickness was 248.71 ± 18.73 µm. A significant age-related reduction in macular thickness was observed, particularly in the inner superior parafovea (p=0.036), nasal perifovea (p=0.001), superior perifovea (p=0.028), outer layer of inferior parafovea (p=0.049), and the inferior perifovea of the full retina (p=0.029). Males showed significantly greater thickness in the outer layer, especially in the outer parafovea (p=0.004) and perifovea (p<0.0001).

**Conclusions:**

This study established normative macular thickness values for healthy older adults in the Brazilian Amazon region using spectral-domain optical coherence tomography. Age and sex were found to significantly influence macular thickness and should be considered when interpreting measurements. These data will support future studies of retinal diseases in this population.

## INTRODUCTION

Optical coherence tomography (OCT) is a non-invasive, rapid imaging tool essential in ophthalmology. It provides *in vivo* images of the retina, optic nerve head, vitreous, and anterior ocular segments, reinforcing its broad role in ocular imaging^(1^,^2)^. OCT is widely used in clinical settings to address diverse conditions^(2)^. Its utility includes diagnosing age-related macular degeneration (AMD), diabetic macular edema, glaucoma, and retinal vascular diseases^(3)^. OCT can assess structural features, such as macular thickness, the retinal nerve fiber layer (RNFL), and the ganglion cell complex. It is vital for diagnosing and monitoring disease severity or progression by comparison with normal population data^(3)^.

Several studies have shown that factors such as ethnicity, sex, age, and equipment type can affect macular and RNFL thickness measurements^(4^-^7)^. For instance, a study of 105 Indian subjects^(8)^ reported a lower mean central macular thickness compared with previously published data from a group containing Black, Asian, and predominantly White healthy subjects^(9)^. Another study found that males typically have greater macular thickness compared with females^(10)^. In comparisons involving two or more OCT devices, variations in central macular thickness have been reported in the 2–77-µm range^(11)^.

The Brazilian Amazon Region Eye Survey (BARES) was a population-based study designed to investigate the prevalence and causes of distance and near visual impairment, as well as blindness, among older adults in Parintins, a city in the Amazon Region^(12)^. This region has distinct geographic, demographic, and sociocultural characteristics, differing from other Brazil areas, particularly the Southeast, which has received more pronounced research attention^(12^,^13)^. Limited health data on this population stems partly from access barriers and logistical challenges faced during the study^(14)^.

The ethnic composition of Amazonian Brazilians reflects a long process of blending that began with indigenous groups and continued with White Europeans (primarily Portuguese), African descendants, and migrants from other Brazilian regions, especially the Northeast during the rubber economic boom^(15^-^17)^. According to the *Instituto Brasileiro de Geografia e Estatística* (IBGE) census, 64.8% of the population in the Brazilian Amazon region identifies as multiracial (“pardo”), 25.1% as White, 7.5% as Black, 1.6% as Indigenous, and 1.1% as Asian^(18^,^19)^.

Research has shown that OCT data can markedly improve accuracy when determining the causes of visual impairment and blindness in various ocular conditions^(20^,^21)^. However, OCT scans are rarely included in population-based eye surveys, such as BARES, owing to their high cost and complex components, which limit portability. Additionally, these devices often rely on normative databases based primarily on healthy White subjects (reported as Caucasians), which do not represent all populations^(22^,^23)^.

This study aimed to use spectral-domain OCT (SD-OCT) to measure macular thickness in the healthy eyes of BARES participants. It also explored potential sociodemographic factors linked to these measurements. The findings may enhance the clinical evaluation of retinal diseases and support diagnosis and treatment monitoring for underserved populations, particularly through future telemedicine protocols.

## METHODS

### Study design and population

BARES was a population-based, cross-sectional study on the prevalence of near and distance vision impairment and blindness in non-institutionalized residents of Parintins, conducted during September 2013-May 2015. OCT was included in the study to help identify the causes of vision impairment and blindness. However, due to logistical and geographic barriers, OCT was performed only on a subset of participants. The study was approved by the Committee on Ethics for Research of *Universidade Federal de São Paulo* and *Universidade Federal do Amazonas* and followed the Declaration of Helsinki guidelines.

Cluster sampling, based on geographically defined census sectors, identified residents aged ≥45 years living in 20 randomly selected clusters (14 urban and 6 rural) in Parintins, located on the margins of the Amazon River, Amazonas State, Brazil. Eligibility was determined via door-to-door surveys, and participants were invited to attend clinical ophthalmic examinations. Inclusion criteria included being at least 45 years old on the day of the household visit and residing in one of the selected clusters for at least the past 6 months. Further details on the study area, eligibility, visual acuity testing, and eye examinations are available elsewhere^(12)^.

The IBGE, defines race considering self-reported skin color including five categories: White, Black, multiracial (mixed White, African, or Indigenous ethnicity), Yellow (Asians), and Indigenous^(24)^.

### Ocular examination

The ophthalmic examination included uncorrected visual acuity and acuity with presenting correction, mea sured using a retro-illuminated logarithm of the minimum angle of resolution (logMAR) tumbling E chart at 4 m. Best-corrected visual acuity (BCVA) was determined after autorefraction, followed by subjective refraction, conducted by an ophthalmologist. Biomicroscopy was performed before and after pupil dilation, focusing on lens status, classified as normal, phakic with cataracts, pseudophakic, pseudophakic with posterior capsule opacity, aphakic, aphakic with posterior capsule opacity, or undetermined. Intraocular pressure (IOP) was measured using applanation tonometry under topical anesthesia after administering 0.5% proxymetacaine hydrochloride eye drops. A fundus examination was also conducted under pupil dilation using 1% tropicamide eye drops.

### Inclusion and exclusion criteria

A subset of BARES participants was selected based on the following inclusion criteria: best-corrected distance visual acuity (BCDVA) of 20/32 or better in each eye, refraction within ±4.0 diopters spherical equivalent, no anterior segment abnormalities based on biomicroscopy observation, and no posterior segment abnormalities in a dilated fundus examination. Additionally, an IOP of ≤21 mmHg was required, as well as no history of ocular surgery. Exclusion criteria included any opacity and a history of glaucoma, diabetes, or ocular trauma.

### OCT images acquisition protocol

SD-OCT scans were performed using the iVue-100 SD-OCT device (Optovue Inc., Fremont, California, USA) after pupil dilation. The system’s image acquisition rate was 25,000 A-scans/s, with frame rates between 256 and 1024 A-scans/frame. Optical resolution was 5 µm in depth. Image quality was assessed using the signal strength index (SSI), which measures reflected light intensity. Scans with poor quality were excluded from analysis. For instance, images with segmentation failures, algorithm errors, motion artifacts, poor focus, lack of centering, or SSI below 50 were omitted.

Macular thickness was measured using standard iVue scanning protocols. The Early Treatment Diabetic Retinopathy Study (ETDRS) subfields were used to assess full retinal thickness across nine regions, including parafoveal, perifoveal, nasal, superior, inferior, and temporal areas. Thickness measurements included both inner retinal boundaries (from the internal limiting membrane to the outer limit of the inner plexiform layer) and outer retinal boundaries (from the outer limit of the inner plexiform layer to the retinal pigment epithelium). In total, 29,174 A-scans were analyzed, with a higher sampling density at the central fovea. The EMM5 protocol consisting of a primary macular mapping scan, was used to generate average foveal thickness (1 mm in diameter, centered on the fovea) as well as full, inner, and outer retinal thickness maps.

### Statistical analysis

Data from one eye per participant were used for analysis, prioritizing the first tested eye or the eye with better centralization and image quality, provided that both eyes had a BCDVA of 20/32 or better. Descriptive statistics were summarized in frequency tables. Associations between macular thickness and age, sex, education (used as a proxy for socioeconomic status), and ethnicity were examined using multiple linear regression. Statistical analyses were performed in Stata Statistical Software Release 14.0, with statistical significance set at p≤0.05.

## RESULTS

**Figure 1:**
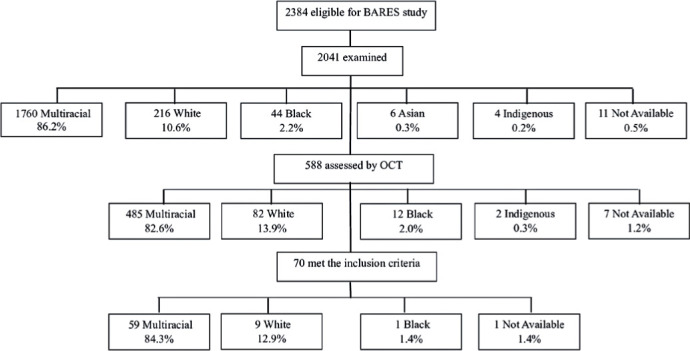
Recruitment flowchart for the normative OCT study, including the distribution of self-reported skin color. The *Instituto Brasileiro de Geografia e Estatística* (IBGE) classifies skin color or race into five self-declared categories: White, Black, multiracial (mixed White, African, or Indigenous ethnicity), Asian, and Indigenous.

In total, 2,384 eligible individuals were identified, with 2,041 (85.7%) undergoing examination and 588 (28.8%) receiving an OCT assessment. Among this subset, 70 (11.90%) met the inclusion criteria for this study. The flowchart in figure 1 illustrates participant selection, showing that approximately 82% were recorded as multiracial at each stage. Of the 70 participants, 45 (64.29%) were female. The age range was 45–65 years, with a mean age of 52.55 ± 5.78 years. Table 1 summarizes the demographic characteristics of participants, including sex, age, education level, and skin color. IOP ranged from 6 to 19 mmHg, with a mean of 12.92 ± 2.44 mmHg. Refractive error in spherical equivalent varied from -1.50 to +2.50 diopters, with a mean of 0.42 ± 0.81 diopters. Supplementary Table 1 lists individual data on sex, age, subjective refraction, and BCVA for each eye.

**Table 1:** Demographic characteristics of participants, including sex, age, education, and self-reported skin color

	n (%)
Sex	
Female	45 (64.29)
Male	25 (35.71)
Age (years)	
45–54	44 (62.86)
55+	26 (37.14)
Education	
<Primary	8 (11.43)
Primary	13 (18.57)
Secondary	17 (24.29)
High school or higher	32 (45.71)
Skin color	
Multiracial	59 (84.28)
White	9 (12.86)
Black	1 (1.43)
Not Available	1 (1.43)
Total	70 (100.0)

### Macular thickness

**Figure 2:**
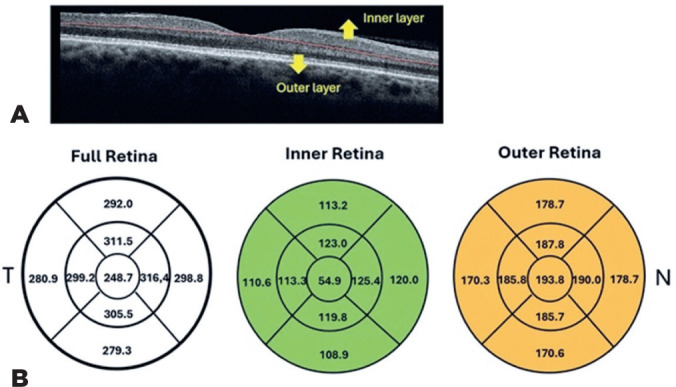
(A) Representative OCT scan from a healthy eye, illustrating segmentation of the retinal layers. (B) Mean macular thickness (in mm) measured across the Early Treatment Diabetic Retinopathy Study (ETDRS) grid for the full retina, inner retina, and outer retina (N: nasal; T: temporal).

Among the 70 participants, central foveal thickness ranged from 222 to 279 µm, with a mean thickness of 248.71 ± 18.73 µm. Figure 2 presents the mean and standard deviation values for all nine ETDRS subfields, organized into full thickness, inner, and outer retinal layers.

A multiple linear regression model, adjusted for age, sex, education, and skin color, was used to identify factors associated with macular thickness. Age showed a negative association with macular thickness in several subfields. In the inner retina, age was negatively associated with the superior parafoveal (p=0.036), nasal perifoveal (p=0.001), and superior perifoveal (p=0.028) subfields. In the outer retina, the inferior parafoveal (p=0.049) and inferior perifoveal full thickness (p=0.029) areas also showed a negative correlation with age.

**Figure 3:**
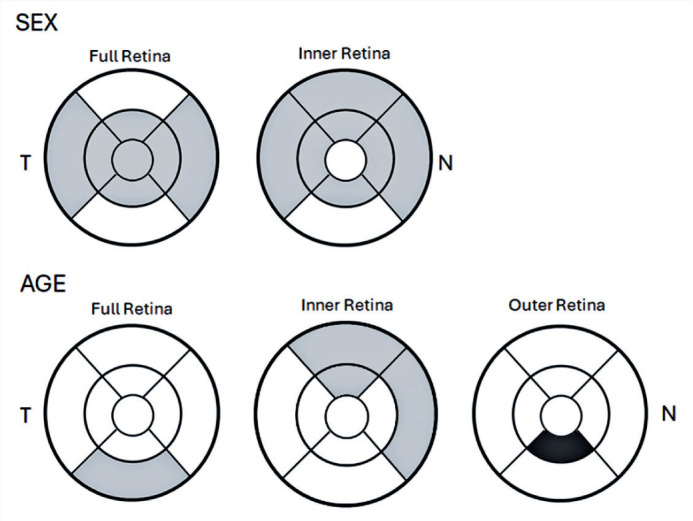
Retinal thickness map showing regions with statistically significant differences by sex and age. Gray areas indicate regions where males and younger participants had thicker measurements. The single black area denotes where older participants had thicker measurements than younger participants.

Macular thickness was significantly greater in males than in females in the central full thickness subfield (p=0.041), across all parafoveal quadrants, and in the nasal and temporal perifoveal full thickness areas. In the outer retina, males had thicker measurements in four parafoveal quadrants and in the nasal, superior, and temporal perifoveal areas. No sex-based differences were observed in the inner retinal layers (Table 2). Figure 3 shows an OCT retinal thickness map highlighting subfields with statistically significant differences by sex and age.

**Table 2:** Multiple linear regression results for macular thickness across ETDRS subfields, adjusted for sex and age.

Thickness, µm		All			Sex			Age		
		Total (±SD) n=70	5% percentile	95% percentile	Male (±SD) n=25	Female (±SD) n=45	p-value*	45–54 (±SD) n=44	55+ (±SD) n=26	p-value*
**Full retina**										
Foveolar		248.71±18.73	222	281	255.52 ± 13.88	244.94 ± 20.10	**0.041**	248.05 ± 20.08	249.85 ± 16.52	0.703
Parafoveal	N	316.37±18.14	284	349	323.60 ± 17.52	312.36 ± 17.38	**0.008**	315.84 ± 17.17	317.27 ± 19.98	0.471
	S	311.46±18.90	269	338	317.92 ± 21.64	307.87 ± 16.36	**0.021**	313.30 ± 16.63	308.35 ± 22.23	0.564
	I	305.50±17.23	272	331	312.00 ± 16.21	301.89 ± 16.88	**0.020**	305.09 ± 18.26	306.19 ± 15.66	0.650
	T	299.21±19.07	264	328	305.28 ± 17.78	295.84 ± 19.12	**0.043**	299.07 ± 19.48	299.46 ± 18.74	0.741
Perifoveal	N	298.81±15.35	276	324	303.96 ± 16.79	295.96 ± 13.87	**0.042**	301.68 ± 14.39	293.96 ± 15.97	0.076
	S	292.01±14.54	267	316	293.96 ± 16.88	290.93 ± 15.24	0.406	293.86 ± 14.62	288.88 ± 14.11	0.252
	I	279.34±15.29	259	298	282.68 ± 12.86	277.49 ± 16.33	0.253	282.34 ± 15.64	274.27 ± 13.50	**0.029**
	T	280.91±16.42	255	306	287.04 ± 15.42	277.51 ± 16.13	**0.013**	281.14 ± 15.63	280.54 ± 18.00	0.803
**Inner retina**										
Foveolar		54.96±7.74	41	68	56.20 ± 7.44	54.27 ± 7.90	0.310	55.34 ± 7.84	54.31 ± 7.69	0.879
Parafoveal	N	125.36±13.10	101	145	126.04 ± 15.38	124.98 ± 11.81	0.530	127.14 ± 12.6	122.35 ± 13.61	0.335
	S	122.97±14.10	100	142	124.04 ± 17.93	11.64 ± 11.63	0.595	126.05 ± 11.39	117.77 ± 16.75	**0.036**
	I	119.79±11.36	99	134	120.76 ± 12.86	10.56 ± 10.56	0.504	121.52 ± 12.27	116.85 ± 9.12	0.164
	T	113.34±11.73	93	132	115.28 ± 13.79	10.42 ± 10.42	0.344	114.66 ± 11.85	111.12 ± 11.40	0.282
Perifoveal	N	120.10±10.46	104	137	119.84 ± 11.68	120.24 ± 9.86	0.933	123.32 ± 90	114.65 ± 10.67	**0.001**
	S	113.23±9.41	101	125	111.64 ± 12.61	114.11 ± 7.06	0.298	115.18 ± 7.84	109.92 ± 10.98	**0.028**
	I	108.88±6.72	98	119	110.28 ± 6.15	108.11 ± 6.96	0.167	110.27 ± 6.82	106.54 ± 5.95	0.067
	T	110.57±9.55	95	123	112.16 ± 10.31	109.69 ± 9.10	0.290	111.80 ± 9.10	108.50 ± 10.11	0.266
**Outer retina**										
Foveolar		193.76±16.37	160	216	199.20 ± 11.04	190.73 ± 18.09	0.068	192.64 ± 17.07	195.65 ± 15.24	0.590
Parafoveal	N	191.00±14.37	173	222	197.48 ± 13.71	187.40 ± 13.57	**0.004**	188.64 ± 11.51	195.00 ± 17.76	0.055
	S	188.37±13.74	165	226	193.80 ± 15.19	12.02 ± 12.02	**0.007**	187.16 ± 11.16	190.42 ± 17.33	0.168
	I	185.66±13.14	169	214	191.12 ± 14.58	11.33 ± 11.33	**0.009**	183.45 ± 10.83	189.38 ± 15.86	**0.049**
	T	185.84±11.70	166	206	190.08 ± 10.52	11.76 ± 11.76	**0.014**	184.34 ± 10.75	188.38 ± 12.97	0.087
Perifoveal	N	178.74±11.62	163	201	184.24 ± 11.66	175.69 ± 10.54	**0.004**	178.41 ± 9.66	179.31 ± 14.55	0.680
	S	178.74±10.19	163	193	182.16 ± 10.89	176.84 ± 9.37	**0.039**	178.68 ± 9.72	178.85 ± 11.15	0.796
	I	170.56±14.40	157	185	172.48 ± 9.03	169.49 ± 16.67	0.573	172.20 ± 16.51	167.77 ± 9.57	0.130
	>T	170.33±9.02	156	186	174.84 ± 7.72	167.82 ± 8.78	**<0.001**	169.36 ± 8.32	171.96 ± 10.07	0.081

N= nasal; S= superior; I= inferior; T= temporal; SD= standard.

Qualitative OCT analysis was performed by an experienced OCT examiner (SESW). OCT abnormalities were detected in six eyes, all with 20/20 BCVA and no findings on clinical examination. As shown in Supplementary Table 1, these included drusen in three eyes (participant #9, right eye; participant #30, both eyes), dry AMD with preserved foveal architecture in both eyes of participant #24, and a serous foveal detachment in the right eye of participant #68.

A sensitivity analysis was conducted to assess the robustness of the observed sex effect on foveolar thickness (p=0.041). Controlling for outliers, performing a jackknife analysis, and testing for age–sex interaction effects showed no change in statistical significance. Although the observed association is statistically robust, confirmation in larger population-based samples is recommended.

## DISCUSSION

This study has several strengths. First, it used a rigorous sampling methodology. Second, data quality was assured by an experienced team of ophthalmologists and researchers. Third, the analysis focused on the unique characteristics of a specific Amazon population, reinforced by the skin color distribution in the study sample compared with the full BARES cohort. Notably, few population-based studies have reported normative macular thickness values, and to our knowledge, this first to do so in a representative Amazon population.

Variability in macular thickness among SD-OCT systems has been well documented. The present study evaluated macular thickness in a healthy subgroup from a population-based cohort in the Brazilian Amazon region. The mean central foveal thickness was 248.71 ± 18.73 µm, comparable to values obtained using the same Optovue OCT system from 168 healthy White adults with a similar mean age in Bulgaria^(26)^ and 45 healthy Hispanic participants with a similar age range in Colombia^(27)^.

Consistent with previous findings^(10^,^28^,^29)^, we found greater macular thickness in males across nearly all regions. As anticipated, macular thickness declined with age in both sexes. All participants had normal BCVA, supporting the exclusion of common vision-impairing conditions, such as cataracts and macular abnormalities. Extensive research on the ETDRS map area has shown that sex and age influence macular thickness. Some studies have associated thicker foveae with male sex and older age^(29^,^30)^, whereas others found that foveal and macular thickness correlate only with male sex^(10)^. In the present study, males exhibited significantly thicker outer retinal layers, whereas thinning of the inner layers, particularly in the nasal parafoveal area, was associated with aging in both sexes. The observed sex-based difference in outer retinal thickness likely reflects structural variation in the photoreceptor and retinal pigment epithelium layers. This aligns with studies showing that males tend to have thicker combined inner nuclear, outer plexiform, and outer nuclear layers^(31)^. Aging is commonly associated with macular thinning^(31^-^33)^, although few studies have specified which layers are most affected^(31^,^33^,^34)^. Prior research using low-resolution OCT 3 equipment showed age-related thinning of the RNFL^(35)^. The current findings support this, particularly in the perifoveal nasal area, i.e., the thickest part of the macular RNFL.

**Table 3:** Supplementary Table 1: Individual-level data, including demographics, refractive error (spherical equivalent), and best-corrected visual acuity (BCVA)

Participant code	Sex	Age	Skin color	Right eye Spherical	Cylinder	Axis	SE	Left eye Spherical	Cylinder	Axis	SE	BCVA OD Snellen	logMAR	BCVA OS Snellen	logMAR
1	M	46	W	-1.5	0	0	-1.50	-1.5	-0.5	90	-1.75	20/20	0.0	20/20	0.0
2	F	49	MR	1.25	0	0	1.25	1.25	0	0	1.25	20/20	0.0	20/20	0.0
3	F	47	MR	1	0	0	1.00	0.75	0	0	0.75	20/20	0.0	20/20	0.0
4	F	45	MR	0	0	0	0.00	0	0	0	0.00	20/20	0.0	20/20	0.0
5	F	48	MR	0	0	0	0.00	0	-0.5	105	-0.25	20/20	0.0	20/20	0.0
6	F	57	MR	3.25	-1	80	2.75	3	-0.75	85	2.63	20/25	0.1	20/25	0.1
7	F	46	MR	1	-0.5	90	0.75	0.5	0	0	0.50	20/20	0.0	20/20	0.0
8	F	62	MR	0.5	0	0	0.50	0.5	0	0	0.50	20/25	0.1	20/32	0.2
9	M	45	MR	2	-0.75	30	1.63	1.75	-0.25	135	1.63	20/20	0.0	20/20	0.0
10	F	53	MR	1.25	0	0	1.25	1	0	0	1.00	20/20	0.0	20/20	0.0
11	F	53	W	0.75	0	0	0.75	0.5	0	0	0.50	20/20	0.0	20/20	0.0
12	F	57	W	2	0	0	2.00	2	0	0	2.00	20/20	0.0	20/20	0.0
13	F	55	MR	0.5	-0.25	105	0.38	1.25	-0.75	85	0.88	20/20	0.0	20/20	0.0
14	F	53	MR	0.75	0	0	0.75	0.75	0	0	0.75	20/20	0.0	20/20	0.0
15	M	60	W	0.75	-0.5	90	0.50	0.5	-0.75	100	0.13	20/20	0.0	20/20	0.0
16	M	55	MR	0	-0.5	98	-0.25	1	0	0	1.00	20/20	0.0	20/20	0.0
17	M	45	MR	0	0	0	0.00	0	0	0	0.00	20/20	0.0	20/20	0.0
18	F	45	MR	0.5	-0.5	35	0.25	0	0	0	0.00	20/20	0.0	20/20	0.0
19	M	51	W	2.25	0	0	2.25	2.5	0	0	2.50	20/20	0.0	20/20	0.0
20	F	52	MR	1.25	0	0	1.25	1.5	0	0	1.50	20/20	0.0	20/20	0.0
21	F	55	MR	0	-0.5	40	-0.25	0	0	0	0.00	20/20	0.0	20/20	0.0
22	F	50	MR	2.75	-1	85	2.25	1.25	0	0	1.25	20/20	0.0	20/20	0.0
23	M	50	W	1.25	0	0	1.25	1.25	0	0	1.25	20/20	0.0	20/20	0.0
24	M	51	W	0	0	0	0.00	0	0	0	0.00	20/20	0.0	20/20	0.0
25	M	48	MR	0	-0.5	90	-0.25	-0.5	-1.5	60	-1.25	20/20	0.0	20/25	0.1
26	F	49	MR	0	0	0	0.00	0	0	0	0.00	20/20	0.0	20/20	0.0
27	M	59	MR	0.5	-0.5	15	0.25	0.25	-0.5	80	0.00	20/20	0.0	20/20	0.0
28	M	62	MR	0	-0.25	90	-0.13	0.25	0	0	0.25	20/20	0.0	20/20	0.0
29	M	56	MR	1	0	0	1.00	0.75	0	0	0.75	20/25	0.1	20/25	0.1
30	M	50	NA	1	0	0	1.00	1	0	0	1.00	20/20	0.0	20/20	0.0
31	M	53	MR	0	-0.5	170	-0.25	0.5	-0.5	80	0.25	20/20	0.0	20/20	0.0
32	F	50	MR	0	0	0	0.00	0	0	0	0.00	20/20	0.0	20/20	0.0
33	F	50	MR	0	0	0	0.00	0.5	-0.5	180	0.25	20/20	0.0	20/20	0.0
34	F	58	MR	2.25	0	0	2.25	2.5	0	0	2.50	20/20	0.0	20/20	0.0
35	F	46	MR	0	0	0	0.00	0.25	-0.25	100	0.13	20/25	0.1	20/20	0.0
36	F	52	MR	1	-0.75	80	0.63	0.5	0	0	0.50	20/20	0.0	20/20	0.0
37	F	62	MR	0.25	-0.25	90	0.13	0.25	0	0	0.25	20/20	0.0	20/25	0.1
38	F	50	MR	1	0	0	1.00	0.5	0	0	0.50	20/20	0.0	20/20	0.0
39	F	48	MR	0	-0.75	95	-0.38	0	0	0	0.00	20/20	0.0	20/20	0.0
40	F	56	MR	-0.25	-0.5	104	-0.50	-0.25	-0.5	67	-0.50	20/20	0.0	20/20	0.0
41	F	49	MR	0.75	0	0	0.75	0.25	0	0	0.25	20/20	0.0	20/20	0.0
42	F	60	MR	0.5	-0.25	75	0.38	0.75	-0.75	150	0.38	20/20	0.0	20/25	0.1
43	F	60	MR	1.75	0	0	1.75	2.25	-1	110	1.75	20/25	0.1	20/20	0.0
44	F	59	MR	0.5	-0.5	75	0.25	0.5	-0.5	95	0.25	20/32	0.2	20/25	0.1
45	F	47	MR	1.75	0	0	1.75	1.5	-0.75	180	1.13	20/20	0.0	20/20	0.0
46	F	47	W	1.75	-0.5	40	1.50	1.75	0	0	1.75	20/20	0.0	20/20	0.0
47	F	46	MR	1	0	0	1.00	0.75	0	0	0.75	20/20	0.0	20/20	0.0
48	F	54	MR	1.75	-1	10	1.25	1.75	-0.5	175	1.50	20/20	0.0	20/20	0.0
49	F	48	MR	1.25	-0.75	180	0.88	1.25	-0.5	180	1.00	20/20	0.0	20/20	0.0
50	M	51	MR	1	0	0	1.00	0.5	0	0	0.50	20/20	0.0	20/20	0.0
51	F	75	MR	1	-2.5	75	-0.25	1.75	-2.5	120	0.50	20/25	0.1	20/25	0.1
52	F	45	MR	0	0	0	0.00	0	0	0	0.00	20/20	0.0	20/20	0.0
53	M	46	MR	1	-0.5	50	0.75	1	-0.75	155	0.63	20/20	0.0	20/25	0.1
54	M	48	MR	0.5	-0.75	85	0.13	0.5	-0.25	125	0.38	20/20	0.0	20/20	0.0
55	M	56	B	2.25	-0.75	65	1.88	2.25	-1	100	1.75	20/20	0.0	20/20	0.0
56	M	48	MR	0.5	-0.5	86	0.25	0	-0.5	96	-0.25	20/20	0.0	20/20	0.0
57	F	57	MR	1	0	0	1.00	1	0	0	1.00	20/20	0.0	20/20	0.0
58	F	57	MR	-1.25	-1	60	-1.75	0.75	-0.5	145	0.50	20/20	0.0	20/20	0.0
59	M	53	MR	0.5	-0.25	60	0.38	-0.25	-0.25	120	-0.38	20/20	0.0	20/25	1.0
60	M	63	MR	0.5	-1.5	20	-0.25	0	-0.75	100	0.00	20/20	0.0	20/20	0.0
61	M	47	MR	0.25	-0.5	85	0.00	0.25	-1	95	-0.25	20/20	0.0	20/20	0.0
62	M	65	MR	2.5	-1	75	2.00	1.75	-1	100	1.25	20/20	0.0	20/20	0.0
63	F	58	MR	1	-0.5	90	0.75	1.5	-0.5	180	1.25	20/25	0.1	20/25	0.1
64	F	47	MR	1	-1	65	0.50	0	0	0	0.00	20/20	0.0	20/20	0.0
65	F	46	MR	0	0	0	0.00	0	0	0	0.00	20/20	0.0	20/20	0.0
66	F	59	MR	1.5	0	0	1.50	1.75	0	0	1.75	20/20	0.0	20/20	0.0
67	F	55	MR	0	0	0	0.00	0	0	0	0.00	20/20	0.0	20/20	0.0
68	F	56	W	1.75	0	0	1.75	2.25	0	0	2.25	20/20	0.0	20/20	0.0
69	M	51	MR	0.5	0	0	0.50	0.5	0	0	0.50	20/20	0.0	20/20	0.0
70	M	53	MR	0.5	0	0	0.50	0.5	0	0	0.50	20/20	0.0	20/20	0.0

OCT-based macular thickness measurements vary by device due to differences in technology, retinal layer boundaries, calibration, algorithms, and image acquisition and processing techniques. Therefore, the present study, focusing on an underrepresented ethnic group, may help establish normative macular layer thickness values, with the added value of age and sex subgroup analyses. Nevertheless, this study has several limitations. First, the age range was 45–65 years, restricting generalizability to other age groups. Second, the sample size was modest (n=70 individuals) compared with larger population-based studies^(30^,^36)^. Third, the study lacked anatomical variables, such as height and axial length, which may influence OCT parameters^(32^,^34^,^37)^. Fourth, the iVue-100 system does not support enhanced depth imaging, preventing analysis of RNFL and choroidal thickness. Finally, logistical barriers to OCT equipment transport in the Amazon Region pose practical challenges.

Despite these limitations, this study provides normative macular thickness data using iVue-100 SD-OCT in healthy adults aged ≥45 years from the Amazon region. These values, derived from a population-based sample, can refine normal OCT reference ranges for this understudied group. These norms may differ from those observed in other populations, such as predominantly White groups typically represented in existing global OCT databases, and improve interpretation of retinal changes, distinguishing normal variation from pathology.

## Data Availability

The datasets generated during and/or analyzed during the current study are available on demand from referees.
